# Three-Dimensional Dense Reconstruction: A Review of Algorithms and Datasets

**DOI:** 10.3390/s24185861

**Published:** 2024-09-10

**Authors:** Yangming Lee

**Affiliations:** RoCAL Lab, Rochester Institute of Technology, Rochester, NY 14623, USA; yangming.lee@rit.edu; Tel.: +1-585-475-4184

**Keywords:** three-dimensional dense reconstruction, deep learning, dataset, robotics, computer vision

## Abstract

Three-dimensional dense reconstruction involves extracting the full shape and texture details of three-dimensional objects from two-dimensional images. Although 3D reconstruction is a crucial and well-researched area, it remains an unsolved challenge in dynamic or complex environments. This work provides a comprehensive overview of classical 3D dense reconstruction techniques, including those based on geometric and optical models, as well as approaches leveraging deep learning. It also discusses the datasets used for deep learning and evaluates the performance and the strengths and limitations of deep learning methods on these datasets.

## 1. Introduction

Three-dimensional dense reconstruction is a computer vision technique that creates a three-dimensional model of an object or scene from a series of two-dimensional images or video frames. The aim is to estimate the 3D geometry and appearance of the object or scene at every point in space, resulting in a detailed and comprehensive 3D model.

Three-dimensional dense reconstruction plays a crucial role in various applications. In robotics, it is essential for tasks such as robot navigation, environment understanding, and object manipulation [1,2,3,4,5,6]. In mixed reality, 3D reconstruction is used to create virtual objects or environments that need to be seamlessly integrated with the real world [7,8,9]. In computer vision, it is employed to generate 3D models for object recognition, tracking, and pose estimation [10,11].

The study of 3D dense reconstruction has a long history, with its origins tracing back to the development of photogrammetry in the mid-19th century [12]. Photogrammetry, the science of making measurements from photographs, is based on principles such as triangulation and stereoscopy [13]. These principles were initially used to create 3D models of terrain and buildings from aerial photographs, leading to rapid growth in the field of photogrammetry during the early 20th century. In the 1970s and 1980s, researchers began developing techniques for reconstructing 3D shapes from multiple images, though these early methods were limited to simple shapes and relied heavily on handcrafted features and heuristics [14,15]. With the advent of digital cameras and the increasing availability of powerful computing resources in the 1990s and 2000s, the field of 3D dense reconstruction experienced significant growth [16]. Researchers introduced new techniques, such as structure from motion [17], multi-view stereo [18], and dense depth estimation [19], enabling more accurate and detailed 3D reconstructions from images. From the early 2010s onward, the evolution of deep learning further advanced 3D dense reconstruction. Researchers developed a variety of new architectures and techniques to predict depth or surface normals from input images, achieving promising results in improving precision, reliability, and applicability.

Datasets are vital for training data-driven deep learning models in 3D dense reconstruction. One of the biggest challenges in using deep neural networks for this task is obtaining high-quality training data. This requires large datasets featuring diverse scenes and objects, captured from various viewpoints and under different lighting conditions. Ideally, these datasets should also be annotated with ground-truth 3D models to train and evaluate the performance of deep neural networks effectively. In recent years, several large-scale datasets, such as the ShapeNet and ScanNet datasets, have been developed specifically for 3D dense reconstruction with deep learning [20,21].

## 2. Geometrical 3D Reconstruction

### 2.1. Overview

The geometry-based 3D dense reconstruction process typically involves the following steps:

1. **Image Acquisition**: The first step is capturing multiple images or video frames of the scene or object from different angles and viewpoints. The quality and resolution of these images are crucial, as they directly impact the accuracy of the final 3D model.
2. **Feature Detection and Matching**: Distinctive features or keypoints are identified within the images, and corresponding features across different images are matched. Common algorithms for this step include SIFT, SURF, and ORB.
3. **Camera Pose Estimation**: Once feature correspondences are established, the relative positions and orientations of the cameras are estimated. This step is essential for reconstructing the geometry of the scene and is typically performed using methods like an essential matrix, homography matrix, or bundle adjustment.
4. **Depth Estimation**: Depth information for each pixel is calculated, often using stereo matching or multi-view stereo techniques. This step generates a dense point cloud that represents the 3D structure of the scene or object.
5. **Surface Reconstruction**: The dense point cloud is then transformed into a 3D mesh that represents the surface of the object or scene. Algorithms such as Poisson surface reconstruction or marching cubes are commonly used in this process.
6. **Texturing**: In the final step, color and texture information from the original images are applied to the 3D mesh, creating a photorealistic 3D model.

In summary, 3D dense reconstruction enables the creation of highly accurate and detailed 3D models from 2D images, offering valuable insights and immersive experiences across various industries and applications.

### 2.2. Structure from Motion

**Figure 1:**
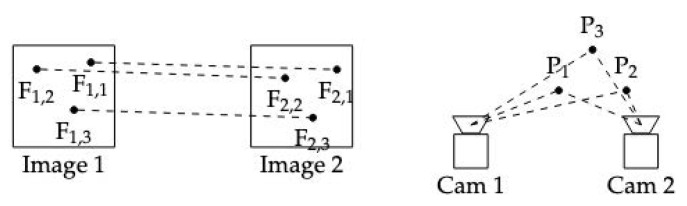
Structure from motion.

Structure from Motion (SfM) is a computer vision technique used to estimate the 3D structure of a scene or object from a series of 2D images or video frames captured from different viewpoints [17]. This process involves estimating the camera’s motion (poses) and the 3D coordinates of scene points by analyzing the apparent movement of object points across the image sequence (Figure 1). SfM is widely applied in fields such as robotics, aerial mapping, and 3D reconstruction.

The typical Structure from Motion (SfM) pipeline closely follows the general 3D reconstruction process. It begins by capturing images or video frames from various angles and then identifies features using algorithms such as SIFT, SURF, or ORB [22,23,24]. These features are matched across images using techniques like nearest neighbor searches or joint compatibility tests [25,26,27]. With the matched keypoints, the pipeline estimates camera positions and orientations using methods like an essential matrix or bundle adjustment, followed by triangulating features to generate a sparse 3D point cloud. For dense reconstruction, the sparse point cloud is interpolated using stereo matching methods, and a 3D mesh is constructed with algorithms like Poisson surface reconstruction [28]. Structure from motion allows for the reconstruction of 3D structures from 2D images while accounting for camera motion, making it a powerful tool for a wide range of applications across various industries.

### 2.3. Shape from Shading

**Figure 2:**
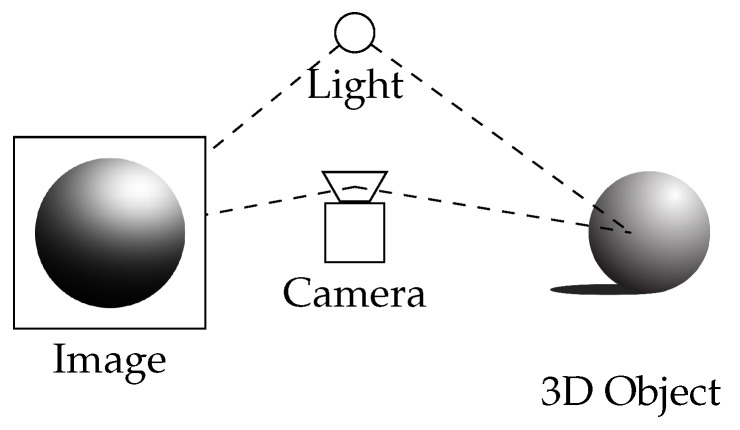
Shape from shading.

Shape from Shading (SfS) recovers the 3D shape or geometry of an object or scene from a single 2D image by analyzing variations in shading or intensity [29]. The shading is influenced by the interplay between surface geometry, illumination, and the material properties of the object (Figure 2). By making specific assumptions about these factors, Shape from shading seeks to infer depth information and reconstruct the 3D structure of the scene.

The assumptions commonly made in Shape from Shading (SfS) include the following:

- **Lambertian Surface**: The object’s surface is assumed to follow a Lambertian reflectance model, reflecting light equally in all directions, with the reflected light intensity depending only on the angle between the light source and the surface normal [29].
- **Known Lighting Conditions**: The position, intensity, and color of the light source(s) are assumed to be known or estimated.
- **Smooth Surface**: The object’s surface is assumed to be smooth, with continuous variations in depth and surface normals.
- **Single Image**: SfS operates on a single image, unlike other 3D reconstruction techniques that rely on multiple images or stereo pairs.

Since Shape from Shading (SfS) utilizes a different approach than Structure from Motion (SfM), its process is notably distinct. The SfS pipeline typically begins with preprocessing the input image to reduce noise and enhance shading information. Next, based on the shading and the assumptions made, surface normals at each pixel are estimated. These estimated normals are then integrated to recover depth information, resulting in a 3D representation of the object or scene. This integration step may involve solving Partial Differential Equations (PDEs), optimization techniques, or other numerical methods [29].

Shape from shading has certain limitations, such as sensitivity to noise, ambiguities in 3D shape reconstruction, and dependence on the assumptions made. Despite these challenges, it remains a valuable technique for 3D reconstruction, particularly when only a single image is available. SfS has been applied in various fields, including robotics, medical imaging, and object recognition.

### 2.4. SLAM

Simultaneous Localization And Mapping (SLAM) is a technique used in robotics and computer vision to simultaneously estimate the camera’s pose (localization) and generate a 3D map of the environment (mapping) [30]. SLAM-based 3D dense reconstruction aims to create a detailed and accurate three-dimensional representation of a scene or object in real time as the camera moves through the environment.

**Figure 3:**
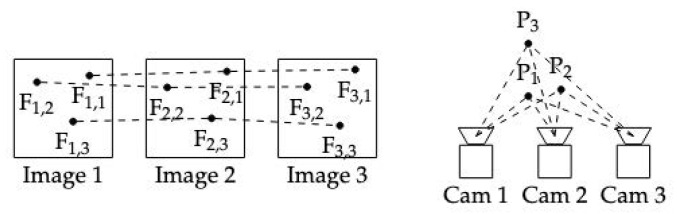
Simultaneous localization and mapping for dense visual reconstruction.

SLAM can be seen as an extension of Structure from Motion (SfM) that incorporates global landmarks [31,32,33]. This is achieved through the continuous identification and updating of global landmarks, often using Bayesian networks or similar techniques. Additionally, loop closing techniques help to minimize error accumulation over time (Figure 3). Some of the commonly used SLAM-based dense reconstruction methods are described below.

KinectFusion is a real-time 3D dense reconstruction algorithm that uses depth data from a Microsoft Kinect sensor [34]. It generates a dense 3D model by continuously fusing depth measurements into a global 3D representation, stored as a Truncated Signed Distance Function (TSDF) volume. The camera’s pose is estimated by aligning the current depth frame with the global model using the Iterative Closest Point (ICP) algorithm.

ElasticFusion combines dense SLAM with non-rigid surface deformation [35]. It builds a global map represented as a surfel-based model while continuously estimating the camera’s pose. This method handles large-scale environments and loop closures, enhancing the consistency of the reconstructed 3D model.

Large-Scale Direct monocular SLAM (LSD-SLAM) is a monocular SLAM method that directly operates on image intensity values, bypassing the need for feature extraction and matching [36]. It estimates the camera’s pose and a semi-dense depth map in real time, producing a 3D model of the environment. LSD-SLAM can also be extended to RGB-D data (color and depth) for more accurate dense reconstructions.

ORB-SLAM is a feature-based SLAM method that uses ORB (Oriented FAST and Rotated BRIEF) features for localization and mapping [24]. Although it primarily creates a sparse 3D map, it can be extended with dense reconstruction techniques, such as stereo matching or multi-view stereo, to generate dense 3D models.

Dense Tracking And Mapping (DTAM) is a monocular SLAM method that generates dense depth maps for each frame in real time by minimizing the photometric error between the input image and a rendered version of the 3D model [37]. These depth maps are merged into a global 3D model, and the camera’s pose is estimated using a dense direct tracking method.

These SLAM-based 3D dense reconstruction methods offer real-time performance and are used in various applications, such as robotics, autonomous navigation, augmented reality, and virtual reality. They excel at handling complex environments and camera motion, making them ideal for generating 3D models in dynamic and real-world scenarios.

## 3. Deep-Learning-Based 3D Dense Reconstruction

Deep learning techniques for 3D dense reconstruction have emerged in response to the limitations of traditional model-based algorithms, which often struggle with adaptiveness and robustness in real-world applications. These advancements are driven by progress in computer vision, graphics, and machine learning.

Currently, deep-learning-based 3D dense reconstruction techniques show notable advantages, including higher accuracy, improved robustness, and scalability. Below is a summary of these algorithms, categorized by the types of techniques that they utilize.

### 3.1. Convolutional Neural Networks

Convolutional Neural Networks (CNNs) are widely used for image-based tasks due to their ability to capture both local and global features, along with their advantages of scale and location invariance [38,39,40,41]. CNNs have been adapted for tasks like depth estimation, surface normal prediction, and semantic segmentation, which can be combined to perform dense 3D reconstruction.

**Figure 4:**
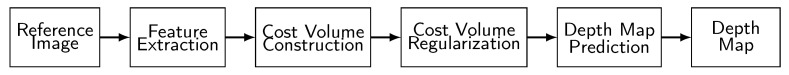
Architecture of deep multi-view stereo.

Deep Multi-View Stereo (DeepMVS) is a representative example of CNN-based dense reconstruction [42]. DeepMVS takes a reference image and neighboring images with known camera parameters as input. Its architecture includes shared-weight CNNs for feature extraction, cost volume construction for similarity calculation, 3D CNN-based cost volume regularization, and depth map prediction using sub-pixel interpolation (Figure 4). Trained end-to-end with a multi-scale loss function, DeepMVS achieves superior performance on various benchmarks.

DenseDepth uses a convolutional neural network to predict depth maps from single RGB images [43]. The model leverages both local and global features to enhance depth estimation accuracy.

Structure from Motion Learner (SfMLearner) is an unsupervised learning framework designed for depth and camera pose estimation from monocular videos [44]. It employs two CNNs: one for depth estimation and another for pose estimation. The model learns depth and ego-motion simultaneously by optimizing the photometric consistency loss between the input image and a synthesized image. SfMLearner does not require ground truth depth or pose annotations, making it suitable for large-scale training on real-world videos.

### 3.2. Three-Dimensional Convolutional Neural Networks (3D-CNNs)

Three-Dimensional Convolutional Neural Networks (3D-CNNs) extend traditional CNNs to operate on volumetric data, making them ideal for tasks like voxel-based representations, point cloud processing, and 3D segmentation.

**Figure 5:**
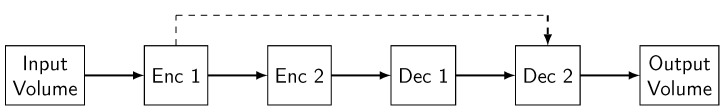
Architecture of 3D UNet.

Volumetric U-Net (3D U-Net) is an adaptation of the popular U-Net architecture for dense 3D segmentation tasks [45]. The network consists of three main components: an encoder, a decoder, and a final layer (Figure 5). The encoder is composed of a series of 3D convolutional layers followed by 3D max-pooling layers, with the number of feature maps doubling after each pooling operation. The decoder uses 3D up-convolutional layers, followed by concatenation with corresponding feature maps from the encoder through skip connections, and then additional 3D convolutional layers. After each up-convolution, the number of feature maps is halved. The final layer is a 1 × 1 × 1 3D convolutional layer that maps the feature maps to the desired number of output channels, corresponding to segmentation classes.

Octree-based CNNs, such as OctNet, utilize an octree representation to efficiently learn 3D structures at high resolutions [46]. This approach is more memory-efficient compared to traditional 3D CNNs but relies on the quality of the octree construction. OctNet can handle sparse annotations and provide accurate reconstructions, though it may require substantial memory and computational resources [45,46].

Voxel-based 3D CNNs, like VoxelNet, convert point clouds into a voxel representation and process them using a 3D CNN for object detection tasks [47]. While effective for detecting objects within point clouds, VoxelNet may be less suited for dense reconstruction tasks compared to octree-based methods.

### 3.3. Recurrent Neural Networks (RNNs) and Long Short-Term Memory (LSTM)

Recurrent Neural Networks (RNNs) and Long Short-Term Memory (LSTM) networks are particularly useful for processing sequences of data, which are common in robotics and can be advantageous for 3D dense reconstruction tasks involving temporal components, such as video-based reconstruction or SLAM [48,49,50,51,52,53]. These networks can model temporal dependencies, enhancing the accuracy of the reconstruction [54]. Existing methods often combine RNNs with other architectures, such as CNNs, to achieve comprehensive 3D dense reconstruction.

**Figure 6:**
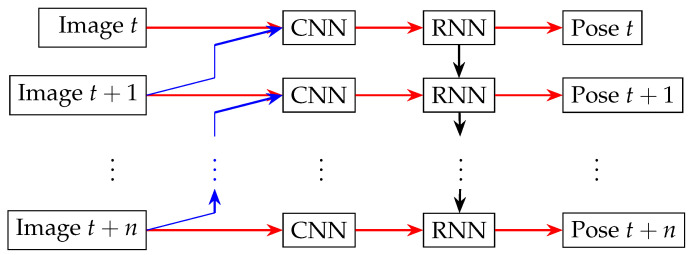
Architecture of deep visual odometry.

Deep Visual Odometry (DeepVO) estimates camera motion and depth from video sequences by integrating CNNs and RNNs [52]. DeepVO comprises two main components: feature extraction and pose estimation (Figure 6). The feature extraction module uses a Convolutional Neural Network (CNN) to process pairs of consecutive monocular images and generate feature maps. This CNN is pretrained on the ImageNet dataset and then fine-tuned for visual odometry. The pose estimation module employs an RNN with LSTM units to predict the six-DOF camera pose from the feature maps. The RNN captures temporal dependencies between consecutive frames, which is crucial for accurate visual odometry.

The Depth and Motion Network (DeMoN) leverages both RNNs and other architectures to estimate depth and camera motion from pairs of images [55]. It consists of two main components: the Depth and Motion Network (DMN) and the Iterative Refinement Network (IRN). The DMN comprises two Convolutional Neural Networks (CNNs) with identical architectures but different weights. The IRN includes several convolutional layers and a Convolutional LSTM (ConvLSTM) module, which is used to model the iterative refinement process. Compared to DeepMVS, DeMoN may offer higher accuracy in estimating depth and motion, although it might be less generalizable [42,55].

Unsupervised Deep Visual Odometry (UnDeepVO) adapts the unsupervised approach of SfMLearner for stereo camera setups, enhancing depth estimation accuracy [56]. However, it requires stereo input data and may still be less accurate compared to supervised techniques.

To summarize, Recurrent Neural Networks (RNNs) excel at handling sequential data and capturing temporal dependencies due to their ability to maintain a hidden state across time steps [57]. This makes them well suited for tasks like time series prediction and natural language processing [53,58]. However, RNNs face challenges such as difficulty in learning long-range dependencies due to vanishing or exploding gradients, and can be computationally intensive [49]. Despite advancements like LSTM and GRU architectures addressing some of these issues, RNNs often require careful tuning and significant computational resources to achieve optimal performance.

### 3.4. Graph Neural Networks (GNNs)

Graph Neural Networks (GNNs) are designed for graph-structured data, making them well suited for processing irregular data structures like point clouds. They have been effectively applied to tasks such as point cloud segmentation, classification, and completion [59].

**Figure 7:**
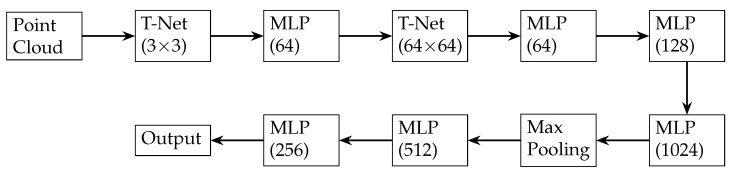
Architecture of PointNet.

PointNet is a deep learning architecture specifically designed for point cloud processing. It utilizes shared multi-layer perceptrons (MLPs) to learn point-wise features and global max-pooling to aggregate these features [60]. The primary goal of PointNet is to learn a global point cloud feature that is invariant to permutations, translations, and rotations. The architecture includes the following five components (Figure 7):

1. **Input**: An unordered set of 3D points, each represented by its x, y, and z coordinates.
2. **Transformation Networks (T-Nets)**: These are mini-PointNets that learn spatial transformations to align the input point cloud. There are two T-Nets: the first predicts a 3 × 3 transformation matrix to align the point cloud and the second predicts a 64 × 64 matrix to align the features.
3. **Multi-Layer Perceptrons (MLPs)**: Fully connected layers that learn local features for each input point. The architecture includes several MLP layers with varying numbers of neurons (e.g., 64, 128, or 1024), applying a shared weight function to each point independently, which ensures permutation invariance.
4. **Max Pooling**: A symmetric function that aggregates local features into a global point cloud feature. Max pooling captures the most salient features of the input point cloud.
5. **Fully Connected Layers and Output (MLPs)**: Processes the global point cloud feature to generate the final output. For classification tasks, the output layer has as many neurons as there are object classes, while, for segmentation tasks, the output layer produces per-point scores.

The Dynamic Graph CNN (DGCNN) constructs dynamic graphs based on nearest neighbors within the input data, enabling the network to adapt to varying point cloud densities [61]. This approach enhances classification and segmentation performance compared to PointNet, although it can be computationally expensive due to the dynamic graph construction.

PointNet++ builds on the original PointNet architecture by applying it hierarchically to capture both local and global features [62]. This hierarchical approach improves performance in dense reconstruction tasks compared to PointNet. However, PointNet++ may be less flexible than DGCNN in handling variations in point densities.

To summarize, GNNs excel at capturing complex relationships and dependencies in graph-structured data, making them highly effective for tasks involving non-Euclidean data like social networks and molecular structures [63]. They can model intricate interactions and dependencies between nodes, enabling advanced applications in areas such as recommendation systems and drug discovery [64]. However, GNNs can be computationally intensive and may struggle with scalability to very large graphs [65]. Additionally, they often require the careful tuning of hyperparameters and can be sensitive to the quality of the graph’s structure, which may affect their performance in practical applications [66].

### 3.5. Generative Adversarial Networks (GANs)

Generative Adversarial Networks (GANs) consist of a generator and a discriminator network that compete with each other, enabling the learning of complex data distributions. GANs have been applied to various tasks, including image synthesis, inpainting, and depth map refinement in dense 3D reconstruction.

**Figure 8:**
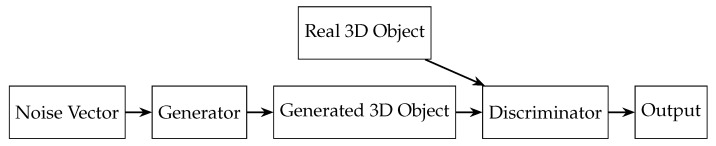
Architecture of 3D generative adversarial network.

The 3D Generative Adversarial Network (3D-GAN) generates 3D shapes by learning a mapping from random noise to 3D volumes [67]. The model comprises two primary components: a generator and a discriminator (Figure 8).

- **Generator**: A 3D Convolutional Neural Network (CNN) that takes a random noise vector as input and produces a 3D object as output. It uses transposed 3D convolutional layers for upsampling, followed by batch normalization and ReLU activation functions. The architecture resembles a 3D U-Net, incorporating skip connections between corresponding layers to refine the generated shapes.
- **Discriminator**: A 3D CNN that classifies the generated 3D object as either real (from the training dataset) or fake (produced by the generator). It consists of several 3D convolutional layers with batch normalization, leaky ReLU activation functions, and a final fully connected layer with a sigmoid activation function.

While 3D-GAN can produce diverse shapes, it may struggle with fine details and accuracy in complex scenes.

Pixel-to-Voxel Reconstruction (Pix2Vox) converts 2D images into 3D voxel representations using a combination of a 3D encoder–decoder network and a 2D encoder network [68]. Designed for single-view reconstruction, Pix2Vox can produce high-quality 3D models. However, it relies on voxel representations, which can be memory-intensive.

### 3.6. Autoencoders and Variational Autoencoders (VAEs)

Autoencoders and Variational Autoencoders (VAEs) are unsupervised learning methods designed to encode and decode data, capturing their underlying structure and distribution. They are used for tasks such as feature learning, denoising, and reconstruction.

**Figure 9:**
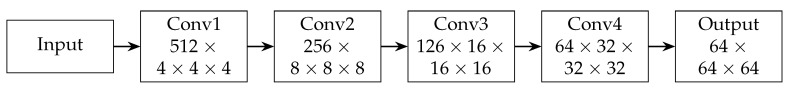
Architecture of the encoder of 3D autoencoder. The decoder has the same architecture.

The 3D autoencoder learns a compact representation of 3D data, including point clouds, meshes, or voxel grids, by encoding and decoding the input data (Figure 9) [67]. The model comprises two primary components:

- **Encoder**: The input to the encoder is typically a 3D representation, such as a voxel grid (3D binary or scalar grid), point cloud (a set of points in 3D space), or a mesh. For voxel-based inputs, 3D convolutional layers are used to capture spatial features from the 3D grid. These layers reduce the spatial dimensions while increasing the depth of the feature maps. In the case of point clouds, layers like PointNet or PointNet++ might be used to extract features directly from the unordered set of points. After the convolutional layers, fully connected (dense) layers are used to further compress the feature representation into a latent space. This latent space is a lower-dimensional representation of the 3D input.
- **Latent Space**: The latent space, also known as the bottleneck layer, contains the compressed representation of the input data. It is typically a vector of fixed size that encodes the most important features necessary for reconstructing the original 3D structure. The size of the latent space is a crucial parameter that balances compression and reconstruction accuracy.
- **Decoder** The decoder begins with fully connected layers that take the latent space vector as input and gradually expand it back to the dimensions of the original 3D representation. For voxel-based inputs, 3D deconvolutional (transposed convolutional) layers are used to upsample the feature maps and reconstruct the 3D structure. These layers progressively increase the spatial dimensions back to the size of the original input. The output layer produces the final 3D reconstruction, typically in the form of a voxel grid, point cloud, or mesh, depending on the original input format.

While 3D autoencoders are a powerful tool for 3D reconstruction, they may require careful tuning and may not be the best choice for all types of 3D data or applications due to their limited ability to generate novel shapes, computational costs, and over-fitting risk.

The 3D Variational Autoencoder (3D-VAE) extends the 3D autoencoder by incorporating a probabilistic layer, which enables the generation of new shapes by sampling from the latent space [69]. This addition provides greater flexibility in generating novel shapes but may result in less accurate reconstructions compared to traditional 3D autoencoders.

Deep neural network architectures, including 3D autoencoders and 3D-VAEs, can be used individually or combined in various ways to achieve the desired level of detail and accuracy in 3D dense reconstruction tasks, depending on specific requirements and available data.

However, the performance of these deep learning models is heavily dependent on the availability and quality of training data. Acquiring well-annotated data for 3D reconstruction can be challenging. Additionally, deep learning models are often considered ‘black boxes’, which can make understanding their inner workings and troubleshooting issues difficult.

### 3.7. Neural Radiance Fields (NeRFs)

Neural Radiance Fields (NeRFs) is a deep-learning-based approach for 3D reconstruction that represents a scene using a continuous volumetric function learned through neural networks. NeRFs model a scene by predicting the color and density of points in a 3D space from multiple input images taken from different viewpoints [70]. This method enables the generation of highly detailed and photorealistic 3D reconstructions.

Using NeRFs for 3D reconstruction has clear advantages:

- High-Quality Reconstructions: NeRFs can produce highly detailed and photorealistic 3D reconstructions by accurately modeling complex lighting and appearance details. This results in high-quality visual outputs that capture fine textures and intricate scene details.
- Continuous Representation: NeRFs represent 3D scenes as continuous volumetric functions, allowing for smooth interpolation and fine details that are challenging to capture with discrete representations like voxel grids.
- View Synthesis: NeRFs excel at synthesizing novel views of a scene, making them effective for applications that require generating images from new viewpoints not included in the training data.
- Flexibility: NeRFs can handle various scene types and can be adapted to different input modalities, such as RGB images and depth maps, enhancing their versatility.

There are also challenges of using NeRFs for 3D reconstruction:

- Computational Cost: Training a NeRF model can be computationally expensive and time-consuming, requiring significant GPU resources and memory. This is due to the need for fine-grained volumetric sampling and the complex nature of the optimization process.
- Data Requirements: NeRFs require a large number of input images from diverse viewpoints to produce accurate and detailed reconstructions. Acquiring and processing these images can be challenging and resource-intensive.
- Inference Speed: While NeRFs generate high-quality reconstructions, the inference process can be slow, as it involves querying the neural network for many points in the volume during rendering.
- Limited Novel Shape Generation: NeRFs are typically trained on existing scenes and may not generalize well to generating novel shapes or objects that were not part of the training data.

Overall, NeRFs provide a powerful and flexible method for 3D reconstruction, delivering high-quality results, especially in well-captured scenes. However, they may not be ideal for all applications due to their computational demands and data requirements [71].

### 3.8. Transformer

Transformers, originally designed for natural language processing, have been adapted for 3D reconstruction tasks due to their ability to handle complex dependencies and long-range interactions [72]. Due to the remarkable learning capabilities demonstrated by transformers in large language models, this approach was quickly adopted in the field of computer vision. By leveraging self-attention mechanisms, transformers can effectively model relationships between different parts of 3D data, leading to improved accuracy and detail in reconstructions.

Transformers for 3D reconstruction use self-attention to weigh the importance of different parts of the input data. This allows them to capture long-range dependencies and complex spatial relationships within 3D data, such as point clouds or voxel grids, improving the quality of the reconstructed 3D models. Transformers can be adapted to various types of 3D data, including point clouds, meshes, and voxel grids. This flexibility allows for more tailored approaches to different 3D reconstruction challenges. Unlike CNNs, which are limited by local receptive fields, transformers can model global context across the entire input. This ability is particularly useful for capturing intricate details and complex structures in 3D reconstruction tasks. Transformers are highly scalable and can handle large and complex datasets, making them suitable for high-resolution 3D reconstruction tasks.

## 4. Dataset for Deep-Learning-Based 3D Dense Reconstruction

A large number of datasets are available for training and testing deep-learning-based 3D dense reconstruction algorithms (Table 1). Each dataset has unique characteristics that make it suitable for different tasks and algorithms. When selecting a dataset for a specific 3D dense reconstruction task, factors such as scene diversity, data quality, and the availability of ground truth data should be considered. For example, datasets like KITTI, SUN3D, and Matterport3D are well suited for evaluating dense reconstruction methods, while Middlebury and ETH3D are widely used for 3D reconstruction and are particularly effective for evaluating stereo and multi-view stereo algorithms. This work will discuss the characteristics of these datasets and compare some of the algorithms, aiming to improve the accuracy and efficiency of dataset selection and benchmark evaluation for researchers.

### 4.1. Dataset Review

ShapeNet (https://shapenet.org/, accessed on 9 July 2024) is a large-scale dataset of 3D models, originally created in 2015 and continually growing [20]. It features over 51,300 unique 3D models categorized into various types, such as chairs, cars, and airplanes. Each model is represented as a collection of 3D meshes, which can be rendered from different viewpoints using a graphics engine to produce 2D images.

Middlebury Stereo Dataset (http://vision.middlebury.edu/stereo/, accessed on 9 July 2024) is a relatively small indoor dataset with high-quality ground truth depth maps acquired using a structured light scanner [73]. It is well suited for benchmarking stereo algorithms and is widely used for evaluating stereo matching performance. The dataset includes an online leaderboard that ranks algorithms based on their performance, with frequent updates as new algorithms are evaluated.

ETH3D (https://www.eth3d.net/, accessed on 9 July 2024) provides both indoor and outdoor scenes with high-resolution images and ground truth depth maps [74]. It features challenging scenes with diverse environments and varying lighting conditions, making it valuable for evaluating multi-view stereo and 3D reconstruction algorithms.

KITTI Vision Benchmark Suite (http://www.cvlibs.net/datasets/kitti/, accessed on 9 July 2024 ) is a comprehensive dataset featuring outdoor street scenes captured with a vehicle-mounted stereo camera rig [75]. It includes high-precision ground truth depth data obtained from LIDAR sensors. However, it is specifically limited to outdoor street scenes.

SUN3D (http://sun3d.cs.princeton.edu/, accessed on 9 July 2024 ) provides RGB-D video sequences of indoor scenes captured using a Microsoft Kinect sensor [76]. The dataset includes camera pose information and a diverse set of scenes, though the depth data, generated by the Kinect, have lower precision compared to LIDAR-based measurements.

Matterport3D (https://niessner.github.io/Matterport/, accessed on 9 July 2024) is a large-scale RGB-D dataset of indoor scenes featuring 10,800 panoramic views from 194 different spaces [77]. It offers high-resolution images, depth maps, and camera poses, covering a diverse range of scenes with various object types and room layouts.

DTU Robot Image Dataset (http://roboimagedata.compute.dtu.dk/, accessed on 9 July 2024) is a multi-view stereo dataset consisting of 60 indoor scenes captured using a robotic-arm-mounted camera [78]. The dataset provides high-resolution images and precise camera poses, focusing on indoor object-centered scenes.

BlendedMVS (https://github.com/YoYo000/BlendedMVS, accessed on 9 July 2024) is a large-scale multi-view stereo dataset with 17,000 high-resolution images of 1000 diverse objects [79]. Its unique feature is the combination of synthetic and real-world images, emphasizing object reconstruction rather than scene reconstruction.

Tanks and Temples (https://www.tanksandtemples.org/, accessed on 9 July 2024) is a dataset featuring both indoor and outdoor scenes with complex geometries and diverse objects [80]. It includes high-quality images and ground truth point clouds generated using a laser scanner.

ScanNet (http://www.scan-net.org/, accessed on 9 July 2024) is a large-scale RGB-D dataset comprising 2.5 million views across more than 1500 indoor scenes [21]. It offers high-quality images, depth maps, and camera poses for indoor environments.

TUM RGB-D Dataset (https://vision.in.tum.de/data/datasets/rgbd-dataset, accessed on 9 July 2024) contains RGB-D video sequences captured with a Microsoft Kinect sensor, along with camera poses obtained from a motion capture system, across various indoor environments [81].

NYU Depth Dataset V2 (https://cs.nyu.edu/~fergus/datasets/nyu_depth_v2.html, accessed on 9 July 2024) features RGB-D data captured using a Microsoft Kinect sensor in indoor environments, including labeled pairs of aligned RGB and depth images [82].

SceneNN (http://www.scenenn.net/, accessed on 9 July 2024) is a comprehensive dataset providing annotated RGB-D images of indoor scenes, along with ground truth annotations for segmentation and 3D reconstruction [83].

Stanford 2D-3D-Semantics (2D-3D-S) (https://3dscenegraph.stanford.edu/, accessed on 9 July 2024) is a dataset containing RGB-D images, 3D point clouds, and semantic annotations for six large-scale indoor spaces. It offers a diverse range of scenes with varying object types and room layouts.

MegaDepth Dataset (http://www.cs.cornell.edu/projects/megadepth/, accessed on 9 July 2024) is a large-scale dataset featuring diverse scenes sourced from the Internet. It includes Internet photos of various scenes and reconstruction results obtained using structure from motion and multi-view stereo techniques [84].

ApolloScape (http://apolloscape.auto/, accessed on 9 July 2024) is a comprehensive dataset for autonomous driving research. It covers semantic segmentation, instance segmentation, 3D car instance reconstruction, and visual localization [85]. The dataset provides high-quality images and annotations across a diverse range of scenes and objects.

OpenRooms (https://vilab-ucsd.github.io/ucsd-openrooms/, accessed on 9 July 2024) is a large-scale dataset featuring synthetic indoor scenes with diverse room layouts. It includes RGB-D images, semantic annotations, and camera poses [86].

Stanford Light Field Archive (http://lightfield.stanford.edu/lfs.html, accessed on 9 July 2024) offers light field images of various scenes and objects, providing unique data for research into light field applications.

Freiburg Forest Dataset (http://deepscene.cs.uni-freiburg.de/, accessed on 9 July 2024) contains RGB, depth, and thermal images captured in a forest environment using a custom sensor setup [87].

3D60: Indoor scene understanding in 3D (https://vcl3d.github.io/3D60/, accessed on 9 July 2024) features 60 indoor scenes with 360° panoramas, depth maps, and semantic annotations [88]. This dataset includes high-quality images and depth maps, making it suitable for evaluating 3D reconstruction, scene understanding, and navigation tasks.

Taskonomy Dataset (http://taskonomy.stanford.edu/, accessed on 9 July 2024) is a large-scale dataset with 4 million images from 20,000 scenes [89]. It includes 3D point clouds, surface normals, and semantic annotations.

Replica Dataset (https://github.com/facebookresearch/Replica-Dataset, accessed on 9 July 2024) provides high-quality 3D reconstructions of indoor environments. It includes RGB-D images, semantic annotations, and camera poses [90].

SCARED Dataset (Stereo Correspondence and Reconstruction of Endoscopic Data) (https://endovissub2019-scared.grand-challenge.org/, accessed on 9 July 2024) is a medical dataset captured using a da Vinci Xi surgical robot. It includes seven training datasets and two test datasets, each corresponding to a single porcine subject, with endoscope observations and camera pose data relative to the robot base recorded based on robot kinematic status [91].

EndoSLAM Dataset (https://github.com/CapsuleEndoscope/EndoSLAM, accessed on 9 July 2024) is a medical dataset featuring both ex vivo and synthetically generated data. The ex vivo part includes standard and capsule endoscopy recordings. The dataset is divided into 35 sub-datasets, specifically 18 for the colon, 5 for the small intestine, and 12 for the stomach [92].

To summarize, SLAM (Simultaneous Localization and Mapping) offers the ability to simultaneously build a map of an environment and track the position of a robot or camera, which makes it highly adaptable to various environments and not reliant on external infrastructure [93]. More importantly, SLAM demonstrates how leveraging redundant information and ensuring consistency can enhance the robustness and practicality of a closed-loop estimation system. This approach of addressing problems by integrating multiple sources of data and maintaining consistency is a valuable strategy that could benefit other methods, such as the widely used end-to-end deep learning systems. Adopting similar principles could improve the reliability and performance of these systems.

### 4.2. Algorithms and Dataset

The performance of deep-learning-based dense reconstruction algorithms is significantly influenced by the quality and size of the dataset used for training. A comprehensive and diverse dataset with precise ground truth depth and camera pose information is essential for effectively training these models.

We provide a summary of the performance of several algorithms on the NYU depth V2 dataset (Table 2), the KITTI vision dataset (Table 3), and the TUM RGB-D dataset (Table 4). This summary illustrates the capabilities of current state-of-the-art techniques. It is important to note that this summary is not intended to be a direct comparison of performance. Rather, it highlights the scale of the challenges that these algorithms address, as they focus on different aspects of 3D dense reconstruction, making direct comparisons less meaningful.

## 5. Discussion

Although deep-learning-based algorithms have achieved significant progress and are nearly dominant in the field of 3D dense reconstruction, they still have several limitations.

These algorithms require large amounts of diverse training data, which can be both time-consuming and costly to acquire. High-quality training data are essential for optimal performance, but obtaining accurate ground truth in scenarios involving depth-sensor denial or time-varying scenes, such as endoscopic surgeries, is nearly impossible [92].

Given these limitations, several challenges remain that necessitate novel and effective solutions:

- **Low Texture**: In scenes with minimal or no texture (e.g., flat, homogeneous surfaces), it becomes challenging to establish feature correspondences between different views. This often results in incorrect depth estimation and can lead to incomplete or noisy reconstructions [108,109,110,111,112,112].
- **Dynamic Objects**: Objects that move between frames or views introduce inconsistencies in the reconstruction process. Depth estimation and correspondence matching typically assume that the scene is static, so dynamic objects can cause errors in the reconstructed geometry [113,114,115,116,117].
- **Low Image Quality**: Images with low resolution, noise, or poor lighting conditions can adversely affect the performance of feature detection and matching algorithms, leading to inaccurate depth estimation and flawed reconstructions. High-quality images are crucial for robust 3D dense reconstruction [118,119,120,121,122].
- **Deformation**: Non-rigid or deformable objects, such as fabric or human bodies, can lead to inconsistencies in the reconstruction process. Deformations may alter an object’s appearance between views, complicating the establishment of correct feature correspondences and accurate 3D structure estimation [123,124,125].
- **Drastic Scene Depth Changes**: Scenes with significant depth variations, such as indoor environments with objects at varying distances, can challenge depth estimation and feature matching. Algorithms must adapt to these variations to achieve accurate reconstructions [110,126,127].
- **Motion Blur**: Fast-moving objects or rapid camera motion can introduce motion blur into images, making it difficult to accurately detect and match features. This can result in incorrect depth estimation and reconstruction artifacts [110,128,129,130].
- **Adverse Illumination Conditions**: Difficult lighting conditions, such as shadows, glare, or over- and under-exposure, can negatively impact feature detection and matching algorithms. Reflective or transparent surfaces may create misleading feature matches due to appearance changes depending on the viewpoint. Robust algorithms need to handle these challenging conditions to ensure accurate reconstruction [131,132,133].

We summarize some of the existing solutions that address one or more of these problem in Table 5.

We believe that several potential solutions can further enhance the robustness, precision, and reliability of 3D dense reconstruction in real-world applications:

Multi-Modal Data Fusion: Combining data from different sensors, such as cameras, LiDAR, and IMUs, can improve the accuracy and reliability of 3D reconstruction. This approach also provides greater robustness against sensor failures or limitations by integrating complementary information from various sources.

Incremental Reconstruction: Incremental reconstruction techniques can enhance the efficiency and robustness of 3D reconstruction in dynamic scenes. Instead of processing the entire scene in one go, incremental methods update the reconstruction as new data become available, allowing for continuous improvement and adaptation.

Incorporating Prior Knowledge: Leveraging prior knowledge about the scene or object being reconstructed can boost the accuracy and robustness of 3D dense reconstruction. This may involve using known object shapes, camera trajectories, or physical constraints such as object rigidity to guide the reconstruction process.

Hybrid Methods: Combining deep-learning-based algorithms with traditional computer vision techniques can improve the overall performance of 3D dense reconstruction. For instance, integrating deep-learning-based feature extraction with conventional structure-from-motion techniques can enhance camera pose estimation accuracy.

Active Learning: Active learning techniques can reduce the amount of labeled data required for training deep learning models. This reduction in data acquisition can lower costs and improve the scalability of the approach.

Causal Deep Learning: We particularly believe that causal deep learning—a relatively new approach focusing on causality in modeling complex systems—holds significant promise. Recent advances in causal deep learning have shown its ability to use prior knowledge to address modeling challenges, reduce data requirements, improve performance on unseen data, modularize learning problems, and enable incremental learning from multiple sources. This approach can fundamentally tackle data scarcity issues and enhance 3D dense reconstruction capabilities [149,150,151].

## Figures and Tables

**Table 1:** Dataset for 3D dense reconstruction.

Dataset	Year of Creation	Size and Type of Scenes	Size	Source of Depth	Camera Pose
ShapeNet	2015	31,350, In & Out	300 M	Synthetic	No
Middlebury Stereo	2001	47, In	47 pairs	Structured light, Stereo	Yes
KITTI Vision	2012	∼28, Out	42,382	Velodyne LiDAR	Yes
ETH3D	2017	27, In & Out	27 sets	Laser scanner	Yes
NYU Depth V2	2012	1449, In	144,959	Kinect	Yes
SUN3D	2013	415, In & Out	N/A	Kinect, Xtion	Yes
TUM RGB-D	2012	39, In	N/A	Kinect	Yes
ICL-NUIM	2014	8, In	N/A	Synthetic	Yes
EuRoC MAV	2016	11, In	N/A	Laser scanner	Yes
ApolloScape	2018	N/A, Out	>140,000	LiDAR	Yes
ScanNet	2017	2513, In	N/A	Kinect v2, RealSense	Yes
Matterport3D	2017	90, In & Out	N/A	Matterport camera	Yes
Stanford 2D-3D-S	2017	6 areas, In	70,496	Matterport camera	Yes
SceneNet RGB-D	2016	5 million, In	5 million	Synthetic	Yes
Sintel	2010	N/A, In & Out	1064	Synthetic	No
Redwood	2016	100, In	N/A	Structure sensor	Yes
FlyingThings3D	2016	N/A, In & Out	3720	Synthetic	Yes
7-Scenes	2014	7, In	N/A	Kinect	Yes
Washington RGB-D	2011	300, In	N/A	Kinect	Yes
Blensor	2013	N/A, In & Out	N/A	Synthetic	Yes
DTU Robot	2014	124, In	5000+	Structured light	Yes
Stanford 3D	2006	N/A, In & Out	N/A	Range scans	Yes
Freiburg Forest	2016	1, Out	N/A	Stereo	Yes
SCARED	2017	7, Med	15,000	Kinect/Synthetic	Yes
EndoSLAM	2016	35, Med	60,000	CT	Yes

Note: In, Out, and Med denotes Indoor, Outdoor, and Medical scenes, respectively.

**Table 2:** NYU depth V2 dataset.

Algorithm	RMSE (m)	Rel Error	σ<1.25	$\sigma <1.25^{2}$	$\sigma <1.25^{3}$
[94]	0.641	0.214	0.611	0.887	0.971
[95]	0.573	0.127	0.811	0.953	0.988
[96]	0.523	0.120	0.838	0.976	0.997
[84]	-	-	0.821	0.965	0.995
[97]	0.471	0.187	0.815	0.955	0.988
[98]	-	-	0.852	0.970	0.994

**Table 3:** KITTI vision dataset.

Algorithm	RMSE (m)	Rel Error	σ<1.25	$\sigma <1.25^{2}$	$\sigma <1.25^{3}$
[99]	6.266	0.203	0.696	0.900	0.967
[100]	4.627	0.117	0.845	0.951	0.984
[101]	4.863	0.187	0.809	0.953	0.986
[102]	4.459	0.115	0.861	0.961	0.986
[103]	4.401	0.112	0.868	0.967	0.991

**Table 4:** TUM RGB-D dataset. RMSE: root mean square error), Acc: accuracy, ATE: absolute trajectory error, and EPE: endpoint error.

Algorithm	Performance
[84]	RMSE: 0.573
[104]	Acc.: 72.34%
[55]	ATE: 0.0177
[105]	ATE: 0.0135
[36]	ATE: 0.0189
[106]	EPE: 0.0163
[107]	ATE: 0.0165

**Table 5:** Example algorithms that address challenges in real-world 3D reconstruction.

	Static	Dynamic Object	Low Texture	Image Quality	Illumination	Recovery	Motion	Deformation	Scene Depth
sparse	[24,134]	[116,135,136,137]	[138,139]	[140]	[35,139]	[141,142]	[143]		
semidense	[144,145]	[146]	[147]	[144]					
full-dense	[148]	[113,114,115]	[108,109,110,111]	[118,120]	[131,132,133]		[110]	[123,124]	[110,126]
